# Energy Drinks Decrease Left Ventricular Efficiency in Healthy Children and Teenagers: A Randomized Trial

**DOI:** 10.3390/s22197209

**Published:** 2022-09-23

**Authors:** Felix Sebastian Oberhoffer, Pengzhu Li, André Jakob, Robert Dalla-Pozza, Nikolaus Alexander Haas, Guido Mandilaras

**Affiliations:** Division of Pediatric Cardiology and Intensive Care, University Hospital, LMU Munich, 81377 Munich, Germany

**Keywords:** energy drinks, pediatrics, echocardiography, left ventricular function, left ventricular dysfunction, randomized trial

## Abstract

Background: Minors are considered the main consumer group of energy drinks (EDs). The aim of this study was to investigate the acute effects of ED consumption on left ventricular (LV) hemodynamics and efficiency in healthy children and teenagers. Methods: This study was a randomized, single-blind, placebo-controlled, crossover clinical trial. Study participants consumed a weight-adjusted amount of an ED or a placebo on two consecutive days. LV hemodynamics and efficiency parameters were evaluated non-invasively by generating LV pressure–volume loops (PVLs) through simultaneous echocardiography and blood pressure measurement. Results: A total of 24 children and teenagers (14.90 ± 2.27 years, 13 male) were included in the present study. Conventional echocardiographic parameters of LV function did not show significant differences between both beverage groups. The non-invasive generation of LV PVLs revealed a significantly lower cardiac efficiency 240 min after the ED consumption compared to the placebo intake (140.72 (133.21–149.73) mmHg vs. 135.60 (124.78–140.33) mmHg, *p* < 0.01). Conclusions: Acute ED consumption is associated with a significantly lower cardiac efficiency in healthy minors. The generation of non-invasive LV PVLs might be beneficial in the assessment of subtle changes in LV efficiency. Further studies need to investigate the influence of chronic ED consumption on LV function and morphology.

## 1. Introduction

Energy drinks (EDs) are sweetened beverages that contain high amounts of caffeine and other stimulants, such as taurine, guarana or glucuronolactone. EDs are particularly popular among children and teenagers, as a survey of the European Food Safety Authority (EFSA) suggests [1]. Accordingly, approximately two-thirds of European adolescents have consumed EDs. Of these, 12% are defined as high acute consumers, drinking over one liter of an ED in a single session [1].

Several pediatric case reports describe adverse cardiovascular events, such as severe cardiac arrhythmia, arterial hypertension or coronary artery dissection, after acute ED consumption [2,3,4]. In addition, excessive and chronic ED consumption is potentially linked with cardiotoxicity and the onset of cardiomyopathy [5,6,7].

While minors are considered the main ED consumer group, systemic studies investigating the acute and chronic effects on the pediatric cardiovascular system are lacking. Recently, a publication of our department revealed a significant increase in systolic (SBP, mmHg) and diastolic blood pressure (DBP, mmHg) of up to 5.23 mmHg and 3.29 mmHg after acute ED consumption in healthy children and teenagers, respectively [8]. As an increase in blood pressure is closely related to an elevation in left ventricular (LV) afterload, it can be assumed that the LV function might be impaired by acute and chronic ED consumption.

To the best of our knowledge, studies evaluating the acute effects of ED consumption on pediatric LV function do not exist. Interestingly, clinical studies with adult subjects revealed a significant increase in LV contractility after acute ED consumption [9,10]. However, the placebo beverages used in these studies either contained caffeine or a different sugar amount [9,10]. In addition, LV function was only assessed at baseline and one hour after ED consumption [9,10]. Hence, the influence of acute ED consumption on LV hemodynamics and efficiency requires further research, particularly in children and adolescents. 

Conventional echocardiographic efficiency parameters of the LV, such as ejection fraction (EF, %) or global longitudinal strain, are considered load-dependent [11]. To overcome load dependency and to assess LV efficiency more precisely, non-invasive imaging modalities are required that incorporate LV afterload [11]. The gold standard for the assessment of LV hemodynamics and efficiency are cardiac-catheterization-derived pressure–volume loops (PVLs) [12]. In the past, validated methods have been established to generate LV PVLs non-invasively by echocardiography and simultaneous blood pressure measurement, allowing for the implementation of LV hemodynamics and efficiency parameters in the ambulatory setting [12,13]. As non-invasively generated LV PVLs consider LV afterload, the deducted LV efficiency parameters seem to be more robust against load dependency. For instance, the generation of non-invasive LV PVLs enables the assessment of the total LV mechanical energy required to generate 1 mL of LV stroke volume (SV, mL) [13].

The aim of this study was to investigate the acute effects of ED consumption on LV hemodynamics and efficiency in healthy children and teenagers by conducting a randomized, single-blind, placebo-controlled, crossover clinical trial.

## 2. Materials and Methods

### 2.1. Ethical Statement

This study was approved by the Ethics Committee of the Ludwig Maximilians University Munich (Munich, Germany) on 12 January 2021 (project number 20-0993). Prior written informed consent was obtained from all study participants, and for minor study participants, consent was additionally obtained from parents or legal guardians.

### 2.2. Study Population

For this study, healthy children and teenagers between the ages of 10 and 18 years were recruited prospectively via public calls within the greater Munich (Germany) area. Prior to enrollment, study participants were screened through a personal interview, clinical examination, transthoracic echocardiography, 24 h Holter ECG and 24 h blood pressure measurement. The exclusion criteria were as follows: presence of chronic conditions (e.g., congenital heart disease, arterial hypertension, presence of severe dysrhythmia), history of sudden cardiac death within the family, known allergies against beverage ingredients, regular use of medication with effects on cardiovascular function, regular use of drugs including smoking and alcohol consumption and pregnancy. In addition, study participants were only included if the M-Mode echocardiography-assessed LV EF was ≥55%. The assessment of weight classification, caffeine consumption behavior and ED consumption behavior was described in recent publications of our department [8,14,15].

### 2.3. Study Design

This study was a randomized, single-blind (study participants), placebo-controlled, crossover clinical trial conducted between April 2021 and October 2021 by the Division of Pediatric Cardiology and Intensive Care, University Hospital, LMU Munich (Munich, Germany). The study was registered in the German Clinical Trials Register (https://www.drks.de/drks_web/, registered on 12 January 2022, identifier: DRKS00027580). Detailed information on the study design, including caffeine abstention, fasting conditions and precise beverage ingredients, are described in recent publications of our department [8,14,15]. In short: Study participants were randomized into one of two intervention phases and received a commercially available caffeinated ED or a placebo drink without conventional ingredients found in EDs (e.g., caffeine, taurine) on two consecutive days. The administered ED amount was bodyweight-adjusted (3 mg of caffeine per kilogram of bodyweight) and corresponded to the maximal daily caffeine intake suggested by the EFSA for healthy minors [16]. The administered placebo amount was matched to the ED’s. The ED and the placebo beverage were similar in sugar content. Beverages were given in an identical and masked drinking bottle at room temperature on each study day. To reduce potential effects of circadian rhythm changes, the beverages were given at similar morning hours on both study days [17]. Study participants were provided a sickbed for each study day and asked to stay in the supine position for the whole study duration to minimize the potential influence of physical activity on the cardiovascular parameters studied. After complete data collection, study participants were asked to guess on what study day the ED beverage was administered to evaluate blinding quality.

### 2.4. Echocardiographic Examination

**Figure 1:**
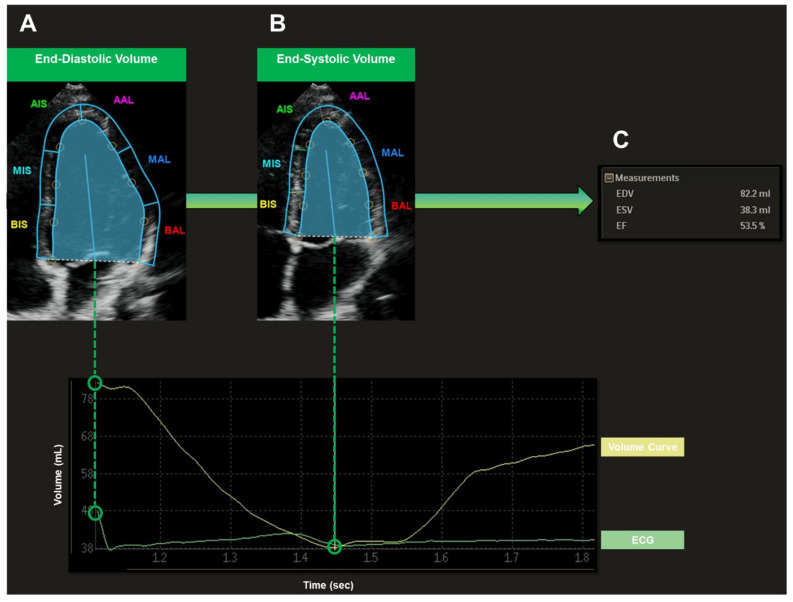
Semiautomatic Assessment of Left Ventricular Volumes Recorded in Apical Four-Chamber View. End-diastole (**A**) (R wave in ECG) and end-systole (**B**) (end of T wave in ECG) were set automatically by the software. The LV endocardium was marked precisely by the investigator in end-diastole (**A**) and end-systole (**B**). The following parameters were then acquired semiautomatically by the software (**C**): EDV, end-diastolic volume; ESV, end-systolic volume; SV, stroke volume; EF, ejection fraction.

For the LV echocardiographic examination, a Philips iE33 xMatrix or a Philips Epiq 7G ultrasound device (Philips Healthcare, Amsterdam, The Netherlands) was used. A 1–5 MHz or a 3–8 MHz sector ultrasound transducer (Philips Healthcare, Amsterdam, The Netherlands) was utilized. The LV was recorded in apical four-chamber view under three-lead ECG tracking. Four consecutive loops were acquired. Special care was taken to portray the entire LV cavity. Recorded loops were transferred to a separate workstation (QLAB cardiovascular ultrasound quantification software, version 11.1, Philips Healthcare, Amsterdam, The Netherlands) for further analysis (Figure 1). End-diastole (R wave in ECG) and end-systole (end of T wave in ECG) were set automatically by the software. The LV endocardium was marked precisely by the investigator in the end-diastole and end-systole. The following parameters were then acquired semiautomatically by the software: LV end-diastolic volume (EDV, mL), LV end-systolic volume (ESV, mL), LV stroke volume (SV, mL), LV ejection fraction (EF, %).

**Figure 2:**
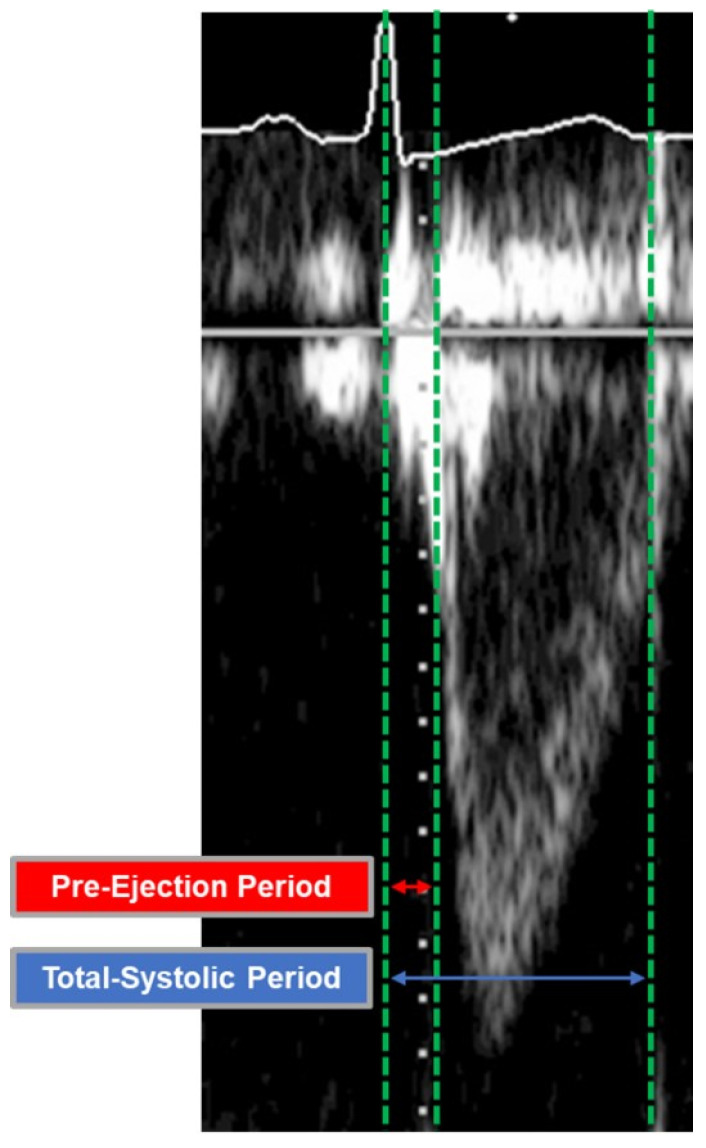
Assessment of the Pre-Ejection and the Total-Systolic Period of the Left Ventricular Outflow Tract. A pulsed wave Doppler was positioned over the aortic valve in apical five-chamber view.

A pulsed wave Doppler was positioned over the aortic valve in apical five-chamber view and under constant three-lead ECG tracking. Recorded clips were then transferred to a separate workstation (IntelliSpace Cardiovascular Ultrasound Viewer, Philips Healthcare, Amsterdam, The Netherlands) to measure the pre-ejection period (R wave in ECG to flow onset, ms) and the total-systolic period (R wave in ECG to end of flow, ms) of the left ventricular outflow tract (Figure 2) [12]. Heart rate (HR, bpm) was calculated automatically by the software.

### 2.5. Blood Pressure Measurement

Blood pressure was measured in supine position with an automated blood pressure device (Infinity M540, Dräger, Germany). Precise information on the assessment of blood pressure was given in a recent publication of our department [8].

### 2.6. Non-Invasive Left Ventricular Pressure-Volume Loops

For the generation of non-invasive LV PVLs, the following parameters assessed via echocardiography and simultaneous blood pressure measurement were required: EDV, ESV, SV, EF, pre-ejection period of the left ventricular outflow tract, total-systolic period of the left ventricular outflow tract, HR, SBP and DBP [13].

#### 2.6.1. Arterial Elastance and End-Systolic Elastance

**Figure 3:**
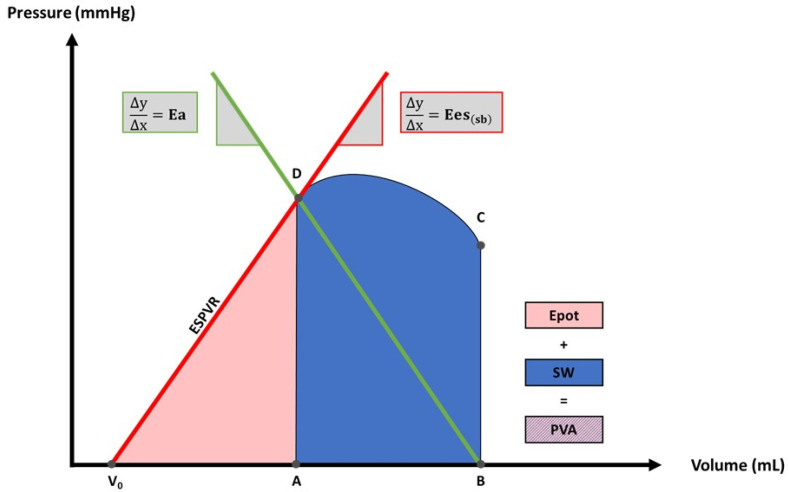
Non-Invasive Left Ventricular Pressure–Volume Loop. ESV, end-systolic volume; EDV, end-diastolic volume; P_es_, end-systolic blood pressure. A (ESV/0 mmHg), B (EDV/0 mmHg), C (EDV/P_es_), D (ESV/P_es_). A–B, diastolic filling; B–C, isovolumic contraction; C–D, ejection; D–A, isovolumic relaxation. ESPVR, end-systolic pressure–volume relationship; Ees_(sb)_, end-systolic elastance derived by single-beat technique; V_0_, intersection between ESPVR and *x* axis; Ea, arterial elastance; Epot, potential energy; SW, stroke work; PVA, pressure–volume area.

Arterial elastance (Ea, mmHg/mL) (slope of the green line in Figure 3) is an indicator of LV afterload and was defined as [13,18,19,20,21]

(1) $$\mathrm{Ea}\;(\frac{\mathrm{mmHg}}{\mathrm{mL}})=\frac{{\;P}_{\mathrm{es}}}{\mathrm{SV}}\;$$

The end-systolic blood pressure (P_es_, mmHg) was calculated as [12]

(2) $${\;P}_{\mathrm{es}}\left(\mathrm{mmHg}\right)=\mathrm{SBP}\times 0.9\;$$

The end-systolic elastance derived by single-beat technique (Ees_(sb)_, mmHg/mL) (slope of the red line in Figure 3), which is considered to be a marker of LV contractility, was calculated in accordance to formulae established by Chen et al. [12].

#### 2.6.2. Potential Energy, Stroke Work and Pressure–Volume Area

The potential energy (Epot, mmHg × mL) is the internal energy that is generated by the LV during systole and “stored” in the ventricular wall [22,23]. Epot needs to be overcome by the heart for LV ejection [23]. Therefore, Epot is not actively involved in LV ejection [13,24]. Epot was calculated as

(3) $$\mathrm{Epot}\;\left(\mathrm{mmHg}\times \mathrm{mL}\right)=\frac{1}{2}\times \left(\mathrm{ESV}-V_{0}\right)\times P_{\mathrm{es}}$$

matching the area enclosed by the end-systolic pressure–volume relationship (ESPVR), the *x* axis and the isovolumic relaxation phase (red area in Figure 3) [13,18,24]. V_0_ is defined as the point of intersection between ESPVR and the *x* axis and was calculated as [13,24]

(4) $$V_{0}=\mathrm{ESV}-\frac{{\;P}_{\mathrm{es}}}{{\mathrm{Ees}}_{\left(\mathrm{sb}\right)}}\;$$

The stroke work (SW, mmHg × mL) represents the effective external work conducted by the LV [24]. SW corresponds to the area enclosed by the PVL (blue area in Figure 3) and was defined as [13,25]

(5) $$\mathrm{SW}\;\left(\mathrm{mmHg}\times \mathrm{mL}\right)=P_{\mathrm{es}}\times \mathrm{SV}\;$$

The pressure–volume area (PVA, mmHg × mL) embodies the total LV mechanical energy generated during one single cardiac cycle and was defined as [13,18,25]

(6) $$\mathrm{PVA}\;\left(\mathrm{mmHg}\times \mathrm{mL}\right)=\mathrm{Epot}+\mathrm{SW}\;$$

#### 2.6.3. Left Ventricular Efficiency Parameters 

The ratio of Ea to Ees_(sb)_ was built to describe ventricular–arterial coupling [13,20]. Cardiac work (CW, mmHg × mL × HR), representing the LV energy expended per minute, was calculated as [13,26]

(7) $$\mathrm{CW}\;\left(\mathrm{mmHg}\times \mathrm{mL}\times \mathrm{HR}\right)=\mathrm{SW}\times \mathrm{HR}\;$$

The efficiency of energy transfer between the LV and the arterial system, also named work efficiency (WE, %), was defined as [13,22]

(8) $$\mathrm{WE}\;\left(\%\right)=\frac{\mathrm{SW}}{\mathrm{PVA}}\times 100\;$$

Cardiac efficiency (CE, mmHg), describing the amount of PVA required to generate 1 mL of SV, was defined as [13]

(9) $$\mathrm{CE}\;\left(\mathrm{mmHg}\right)=\frac{\mathrm{PVA}}{\mathrm{SV}}\;$$

### 2.7. End-Point Measurement

The primary end points were EDV, ESV, SV, EF, Ea, Ees_(sb)_, Epot, SW, PVA, Ea/Ees_(sb)_, CW, WE and CE. End points were assessed at baseline as well as at 30, 60, 120 and 240 min after beverage consumption on each study day (5 measurements on day of ED intake, 5 measurements on day of placebo intake).

### 2.8. Statistical Analysis

As this study was a pediatric pilot study, a prior power analysis was not feasible. Baseline parameters were tested using a paired t-test to determine if the differences between ED and placebo group were normally distributed. The Wilcoxon signed-rank test was chosen if the differences were not normally distributed. To assess the effect of different beverages on the primary end points over time, a two-way repeated-measures ANOVA was performed for normally distributed data (R version 4.1.1). If data were not normally distributed, Ln, Lg or Sqrt data transformation were utilized, and the Wilcoxon signed-rank test was applied. A masked researcher performed data analysis independently. A *p*-value < 0.05 was considered statistically significant.

## 3. Results

In total, 27 healthy children and teenagers were recruited. Of those, three subjects were excluded due to insufficient data quality. Participants’ characteristics are demonstrated in Table 1. None of the subjects had a chronic health condition or were under medical treatment. A total of 13 of the 24 participants (54.17%) correctly guessed the day of ED administration, suggesting appropriate blinding quality. Parameters of LV hemodynamics and efficiency derived by non-invasive PVL generation displayed no significant differences between both groups at baseline (Table 2).

### 3.1. Acute Effects of Energy Drinks on Left Ventricular Hemodynamics

For EDV, non-normal distributions were revealed at time 60 within the ED group and at time 240 within the placebo group. Therefore, the original EDV data were transferred into Sqrt form. For ESV, non-normal distributions were shown at time 60 within the ED group and at times 60 and 240 within the placebo group. Hence, the original ESV data were transferred into Ln form. SV data were normally distributed for all time points for each beverage. For EF, non-normal distributions were revealed at times 30 and 120 within the ED group. The original EF data were transferred into Sqrt form.

Non-normal distributions were revealed for Ea at times 30 and 60 within the ED group and at times 0, 60, 120 and 240 within the placebo group. To achieve normal distribution, the original Ea data were transferred into Lg form. For Ees_(sb)_, an extreme outlier was identified. After outlier exclusion, the statistical conclusions remained the same compared to the original data. For Epot, non-normal distributions were revealed at all time points except at time 0 within the ED group. Hence, the original Epot data were transferred into Lg form, resulting in the identification of an extreme outlier. After outlier exclusion, the statistical conclusions remained consistent. The original SW data were transferred into Sqrt form as non-normal distributions were observed at time 240 within both groups. For PVA, non-normal distributions were demonstrated at times 30 and 240 within the ED group and at times 120 and 240 within the placebo group. Hence, the original PVA data were transferred into Sqrt form, resulting in the identification of two extreme outliers. After outlier exclusion, the statistical conclusion remained consistent.

LV hemodynamics did not differ significantly after the ED and the placebo consumption in study participants (Table 3).

### 3.2. Acute Effects of Energy Drinks on Left Ventricular Efficiency

**Figure 4:**
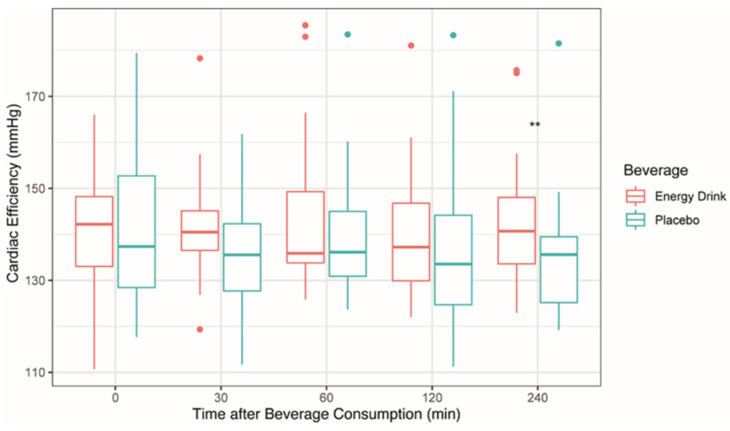
Acute Effects of Energy Drinks on Cardiac Efficiency. ** *p* < 0.01.

For Ea/Ees_(sb)_, WE and CE, the Wilcoxon signed-rank test was applied for each time point as a normal distribution was not able to be achieved through data transformation. For CW, non-normal distributions were revealed at times 30 and 240 within the ED group and at times 30 and 120 within the placebo group. Therefore, the original CW data were transferred into Lg form. Ea/Ees_(sb)_, CW and WE did not differ significantly after the ED and the placebo consumption in study participants (Table 4). Compared to the placebo intake, CE was significantly lower 240 min after ED consumption in study participants (Table 4 and Figure 4).

## 4. Discussion

To the best of our knowledge, this is the first pediatric study investigating the acute effects of ED consumption on LV hemodynamics and efficiency. In total, 24 healthy children and teenagers were included in the present study. Conventional echocardiographic parameters, such as SV and EF, did not display significant differences after the consumption of both beverage groups. When analysing LV efficiency parameters deducted through the generation of non-invasive PVLs, a significantly lower CE was demonstrated after the ED consumption.

### 4.1. Pathophysiological Considerations

The lower CE might be partially explained by the high caffeine amounts contained in EDs. Caffeine is vasoconstrictive and thus leads to an increase in blood pressure [27]. In a similar pediatric cohort, our department revealed a significantly higher SBP, DBP and arterial stiffness after acute ED consumption [8,15]. An elevation in blood pressure and thus LV afterload is associated with an increase in Epot, SW and ultimately PVA to maintain SV and EF. In this study, PVA increased more profoundly after the ED consumption compared to after placebo consumption but did not reach statistical significance.

Caffeine and taurine are considered to increase LV inotropy and hence LV contractility [9,27]. In this study, conventional markers of LV inotropy, such as SV and EF, did not differ significantly between beverage groups. Compared to the placebo intake, Ees_(sb)_ tended to be higher after the ED consumption but did not reach statistical significance. Interestingly, a recent publication of our department revealed that the acute ED consumption is linked with a significantly lower heart rate in children and teenagers [14]. These findings need to be taken into account, as a decrease in heart rate is related to a lower LV contractility [28]. In contrast, studies examining adult subjects revealed a significantly increased LV contractility one hour after ED consumption [9,10]. Doerner et al. investigated LV contractility in 32 healthy adults through cardiac magnetic resonance imaging before and one hour after ED consumption (ED amount: 168 mL/m^2^ body surface area; caffeine concentration: 30 mg/100 mL) [9]. The ED intake led to a significant increase in LV peak systolic strain and the LV peak strain rate [9]. Interestingly, these findings were not demonstrated in ten randomly chosen volunteers when a placebo with a similar caffeine amount was given [9]. Another study, conducted by Menci et al., examined myocardial function in 35 healthy adult volunteers via echocardiography before and one hour after ED consumption (ED amount: 168 mL/m^2^ body surface area; caffeine concentration: 30 mg/100 mL) [10]. The authors revealed a significant increase in mean relative values for mitral annular plane systolic excursion, LV global longitudinal strain and LV twisting after ED intake [10]. However, as these studies examined adult subjects and applied a different study design as well as different imaging modalities, precise comparability with the results demonstrated in the current manuscript is not feasible [9,10].

The acute vasoactive effects of caffeinated EDs are potentially more profound than their inotropic ones. To maintain SV and EF despite increased LV afterload, more LV mechanical energy might be required after the acute ED consumption, ultimately leading to a significantly lower CE.

### 4.2. Energy Drinks: A Potential Threat for Pediatric Heart Function?

This study proposes that acute ED consumption is associated with a significantly lower CE. However, the effects of chronic ED consumption on LV function and morphology remain uncertain. Chronic ED consumption might cause unphysiological blood pressure spikes, the onset of arterial hypertension and increased LV afterload, which could ultimately lead to LV hypertrophy. A chronic decrease in CE due to regular ED consumption could result in a higher myocardial oxygen demand, potentially aggravating the process of LV hypertrophy [29]. Interestingly, several case reports suggest a possible link between chronic ED consumption and the onset of cardiomyopathies [5,6,7]. Minors with increased risk of developing LV dysfunction and/or hypertrophy (e.g., arterial hypertension, aortic valve disease, coarctation of the aorta) should particularly be discouraged from ED consumption. All children and teenagers should be informed about the health risks associated with excessive ED consumption and responsible ED consumption behaviors. Countries such as Lithuania or Latvia have already banned ED sales to minors due to negative health concerns [30,31]. The results of the EDUCATE-Study can be considered as important, as they provide policy makers with long-needed evidence on the cardiovascular effects of acute ED consumption in children and adolescents [8,14,15].

### 4.3. Limitations

#### 4.3.1. Study Design

To the best of our knowledge, this is the first pediatric study investigating the acute effects of EDs on LV function in healthy children and teenagers. Nonetheless, the present study demonstrates certain limitations, some of which have already been discussed in previous publications of our department [8,14,15]. The study sample of the current study was relatively small. In addition, the single-blind (study participants) study design might have led to some bias. However, the offline analysis of echocardiographic loops was executed by a masked investigator. The blinding quality can be considered as adequate, as only 54.17% of study participants correctly identified the day of ED administration. Special care was taken that both beverages were similar in taste and provided in an identical drinking bottle to minimize beverage identification. However, some study participants may have potentially recognized the beverages by taste, smell or physical response. In this study, only one ED product was utilized. The cardiovascular system of minors might react differently to larger ED amounts, different ED products, to the combination of EDs with drugs (e.g., alcohol) and if cardiovascular diseases are already present (e.g., arterial hypertension, rhythm anomalies, congenital heart disease). Moreover, the potential bias due to prior fasting conditions, habitual caffeine effects and interindividual responses of blood pressure to caffeine, needs to be addressed [8,32]. In contrast to previous ED studies in adult subjects, this study did not apply a washout period of several days between the study days [9,17]. Caffeine plasma half-lives are suggested to range between 2.5 and 5 h if given in a single dose of 4 mg per kilogram of bodyweight [33]. However, caffeine clearance can display substantial interindividual variability with half-lives ranging from 2.3 to 9.9 h [33]. Even though baseline parameters did not differ significantly, it cannot be ruled out that some study participants who consumed the ED beverage on the first study day did potentially not fully eliminate caffeine from their systems prior to receiving the placebo beverage. Lastly, the effects on LV efficiency due to chronic ED consumption remain uncertain. As this study revealed a significantly lower CE after the acute ED consumption, further research is required investigating the effects of chronic ED intake on LV function (e.g., manifested LV diastolic and/or systolic dysfunction) and morphology (e.g., LV hypertrophy) in pediatric as well as in adult subjects.

#### 4.3.2. Methodology

The echocardiographic assessment and the offline analysis of the recorded loops can be considered as user-dependent. For a more reproducible measurement of LV parameters, cardiac magnetic resonance imaging might be superior [34]. As LV function was evaluated at multiple time points, the use of cardiac magnetic resonance imaging was thought to be unsuitable for the present study design. EDV and EF were potentially underestimated as LV volumes were assessed semiautomatically in an apical four-chamber view for both study days [35]. However, this method has been proposed to be a quick and accurate tool for LV functional assessment with low intra- and interobserver variability, making it suitable for the present study protocol [35]. The non-invasive calculation of Ees_(sb)_ was only validated in adult subjects; however, high accuracy for pediatric cohorts was demonstrated in the literature [36]. To the best of our knowledge, pediatric reference values of non-invasive PVL-derived LV efficiency parameters do not exist yet and could not be incorporated in the present study.

## 5. Conclusions

Acute ED consumption is associated with a significantly lower LV efficiency in healthy children and teenagers. Moreover, the generation of non-invasive LV PVLs might be beneficial in the assessment of subtle changes in LV hemodynamics and efficiency as no significant differences in LV function were shown through conventional echocardiographic parameters. Further studies need to investigate the influence of chronic ED consumption on LV function and morphology in pediatric as well as in adult subjects. Children and teenagers should be discouraged from drinking EDs, in particular those with pre-existing cardiovascular conditions.

## Figures and Tables

**Table 1:** Study Participants’ Characteristics (*n* = 24).

Characteristics	Total
Age, years (mean ± standard deviation)	14.90 ± 2.27
Sex, *n* (%)	
Male	13 (54.17)
Female	11 (45.83)
Weight Classification, *n* (%)	
Normal weight	21 (87.50)
Overweight	3 (12.50)
Obese	0 (0)
Caffeine Consumption Behavior, *n* (%) ^a^	
Rarely	15 (62.5)
Occasionally	3 (12.5)
Frequently	4 (16.67)
Daily	2 (8.33)
Energy Drink Consumption Behavior, *n* (%) ^b^	
Never	10 (41.67)
Rarely	11 (45.83)
Occasionally	1 (4.17)
Frequently	2 (8.33)
Daily	0 (0)

^a^ Participant was a rare caffeine consumer if they consumed <1 caffeine-containing drink per month, an occasional caffeine consumer if they consumed 1 to 3 caffeine-containing drinks per month, a frequent caffeine consumer if they consumed 1 to 6 caffeine-containing drinks per week and a daily caffeine consumer if they consumed ≥1 caffeine-containing drink per day [17]. ^b^ Participant was a rare energy drink (ED) consumer if they consumed <1 ED per month, an occasional ED consumer if they consumed 1 to 3 EDs per month, a frequent ED consumer if they consumed 1 to 6 EDs per week and a daily ED consumer if they consumed ≥1 ED per day.

**Table 2:** Left Ventricular Hemodynamics and Efficiency Parameters at Baseline (*n* = 24).

Parameters	Energy Drink	Placebo	*p*-Value
EDV (mL)	88.66 ± 20.47	86.83 ± 18.81	0.42
ESV (mL)	46.56 ± 11.82	45.70 ± 11.27	0.67
SV (mL)	42.10 ± 11.41	41.13 ± 10.81	0.61
EF (%)	47.51 ± 6.47	47.21 ± 6.29	0.87
Ea (mmHg/mL)	2.43 (2.09–3.25)	2.58 (2.12–2.98)	0.56
Ees_(sb)_ (mmHg/mL)	3.50 ± 0.93	3.63 ± 1.14	0.49
Epot (mmHg × mL)	1545 (1319–1820)	1442 (1167–1976)	0.66
SW (mmHg × mL)	4301 ± 1266	4222 ± 1188	0.80
PVA (mmHg × mL)	5892 ± 1680	5855 ± 1751	0.32
Ea/Ees_(sb)_	0.75 (0.67–0.82)	0.69 (0.64–0.82)	0.56
CW (mmHg × mL × HR)	290434 ± 82889	288475 ± 75661	0.75
WE (%)	72.77 (70.84–75.04)	74.27 (70.95–75.82)	0.71
CE (mmHg)	139.83 ± 12.59	141.91 ± 15.99	0.49

Mean ± standard deviation is used for normally distributed variables. Median (interquartile range) is used for non-normally distributed variables. EDV, end-diastolic volume; ESV, end-systolic volume; SV, stroke volume; EF, ejection fraction; Ea, arterial elastance; Ees_(sb)_, end-systolic elastance derived by single-beat technique; Epot, potential energy; SW, stroke work; PVA, pressure–volume area; Ea/Ees_(sb)_, ventricular–arterial coupling; CW, cardiac work; HR, heart rate; WE, work efficiency; CE, cardiac efficiency. For SW, PVA and CW, data were normally distributed while the differences between ED and placebo group were not normally distributed. Therefore, the Wilcoxon signed-rank test was applied. For Ea, Epot, Ea/Ees_(sb)_ and WE, data were not normally distributed, while the differences between ED and placebo group were normally distributed. Therefore, the paired t-test was applied.

**Table 3:** Acute Effects of Energy Drinks on Left Ventricular Hemodynamics (*n* = 24).

Parameters	Energy Drink	Placebo	*p*-Value
EDV (mL)	87.93 ± 19.12	88.95 ± 19.78	0.44
ESV (mL)	44.94 ± 11.54	45.51 ± 11.89	0.52
SV (mL)	42.99 ± 10.51	43.43 ± 10.74	0.68
EF (%)	48.98 ± 6.36	48.84 ± 6.31	0.86
Ea (mmHg/mL)	2.56 ± 0.62	2.47 ± 0.67	0.19
Ees_(sb)_ (mmHg/mL)	3.66 ± 1.00	3.47 ± 0.98	0.05
Epot (mmHg × mL)	1624 ± 633	1630 ± 671	0.88
SW (mmHg × mL)	4501 ± 1266	4413 ± 1262	0.40
PVA (mmHg × mL)	6124 ± 1796	6043 ± 1821	0.53

Data are given as mean ± standard deviation. As the statistical conclusions remained consistent before and after data transformation, Table 3 only presents the original data. EDV, end-diastolic volume; ESV, end-systolic volume; SV, stroke volume; EF, ejection fraction; Ea, arterial elastance; Ees_(sb)_, end-systolic elastance derived by single-beat technique; Epot, potential energy; SW, stroke work; PVA, pressure–volume area.

**Table 4:** Acute Effects of Energy Drinks on Left Ventricular Efficiency (*n* = 24).

Parameters	Energy Drink	Placebo	*p*-Value
Ea/Ees_(sb)_			
Time 0	0.75 (0.67–0.82)	0.69 (0.64–0.82)	0.56
Time 30	0.66 (0.64–0.70)	0.66 (0.61–0.75)	1.00
Time 60	0.66 (0.62–0.76)	0.69 (0.61–0.79)	0.87
Time 120	0.65 (0.61–0.74)	0.67 (0.63–0.80)	0.13
Time 240	0.67 (0.62–0.79)	0.71 (0.62–0.75)	0.56
CW (mmHg × mL × HR)	307,145 ± 98,350	304,361 ± 97,313	0.69
WE (%)			
Time 0	72.77 (70.84–75.04)	74.27 (70.95–75.82)	0.71
Time 30	75.12 (74.07–75.84)	75.19 (72.70–76.52)	0.66
Time 60	75.08 (72.58–76.33)	74.28 (71.59–76.50)	0.79
Time 120	75.49 (73.07–76.54)	74.97 (71.38–76.14)	0.15
Time 240	75.05 (71.68–76.22)	73.85 (72.80–76.49)	0.63
CE (mmHg)			
Time 0	139.83 ± 12.59	141.91 ± 15.99	0.49
Time 30	141.86 ± 11.98	137.72 ± 12.38	0.13
Time 60	135.89 (133.12–154.15)	136.15 (129.31–146.06)	0.14
Time 120	137.23 (129.91–146.89)	133.56 (122.90–145.29)	0.21
Time 240	140.72 (133.21–149.73)	135.60 (124.78–140.33)	<0.01 **

Mean ± standard deviation is used for normally distributed variables. Median (interquartile range) is used for non-normally distributed variables. Ea/Ees_(sb)_, ventricular–arterial coupling; CW, cardiac work; HR, heart rate; WE, work efficiency; CE, cardiac efficiency. ** *p* < 0.01. For Ea/Ees_(sb)_, data at times 0, 60 and 240 were not normally distributed, while the differences between ED and placebo group were normally distributed. Therefore, the paired t-test was applied. For Ea/Ees_(sb)_, data as well as the differences between ED and placebo groups at times 30 and 120 were not normally distributed. Therefore, the Wilcoxon signed-rank test was applied. As the statistical conclusions remained consistent before and after data transformation, CW is only presented as orginal data. For WE, the differences between ED and placebo group were normally distributed at times 0, 30, 60 and 240. Therefore, the paired t-test was applied. For CE, the differences between ED and placebo groups were normally distributed at all time points. Therefore, the paired t-test was applied.

## Data Availability

The data presented in this study are available upon reasonable request from the corresponding author.
